# Partial Loss of Function ABCA12 Mutations Generate Reduced Deposition of Glucosyl-Ceramide, Leading to Patchy Ichthyosis and Erythrodermia Resembling Erythrokeratodermia Variabilis et Progressiva (EKVP)

**DOI:** 10.3390/ijms241813962

**Published:** 2023-09-11

**Authors:** Alessandro Terrinoni, Gabriele Sala, Ernesto Bruno, Consuelo Pitolli, Marilena Minieri, Massimo Pieri, Alessandra Gambacurta, Elena Campione, Riccardo Belardi, Sergio Bernardini

**Affiliations:** 1 Department of Experimental Medicine, University of Tor Vergata, Via Montpellier 1, 00133 Rome, Italy; gabrielesala88@gmail.com (G.S.); minieri@med.uniroma2.it (M.M.); massimo.pieri@uniroma2.it (M.P.); gambacurta@med.uniroma2.it (A.G.); belardiriccardo92@gmail.com (R.B.); bernards@uniroma2.it (S.B.); 2 Department of Clinical Sciences and Translational Medicine University of Tor Vergata, Via Montpellier 1, 00133 Rome, Italy; bruno.ernesto@libero.it; 3 Department of Neuroscience, Section of Human Anatomy, Catholic University of the Sacred Heart, Largo Francesco Vito 1, 00168 Rome, Italy; consuelo.pitolli@unicatt.it; 4 Department of System Medicine, University of Tor Vergata, Via Montpellier 1, 00133 Rome, Italy; campioneelena@hotmail.com

**Keywords:** ichthyosis, hyperkeratosis, harlequin ichthyosis, ABCA12, glucosyl-ceramide, EKVP, erythroderma

## Abstract

Ichthyoses are genetically determined cornification disorders of the epidermis characterized by the presence of different degrees of scaling, hyperkeratosis, and erythroderma often associated with palmoplantar keratoderma. Different classifications of these diseases have been proposed, often based upon the involved genes and/or the clinical presentation. The clinical features of these diseases present some overlap of phenotypes among distinct genetic entities, depending mainly on the penetrance of mutations. In this study, using a clinical, genetic, and molecular approach, we analyzed a family with two affected members who had clinical and histological features resembling erythrokeratodermia variabilis (EKV) or a type of erythrodermic hyperkeratosis with palmoplantar keratoderma. Despite of the clinical presentation, we demonstrated that the affected patients were genetically double heterozygous for two different mutations in the *ABCA12* gene, known to be responsible for harlequin ichthyosis. To explain the mild phenotype of our patients, we performed a molecular characterization of the skin. In the upper layers of the epidermis, the results showed a patchy presence of the glucosyl-ceramides (GlcCer), which is the lipid transported by ABCA12, fundamental in contributing to skin impermeability. Indeed, the two mutations detected do not completely abolish ABCA12 activity, indicating that the mild phenotype is due to a partial loss of function of the enzyme, thus giving rise to an intermediate phenotype resembling EKVP, due to a partial depletion of GlcCer deposition.

## 1. Introduction

Ichthyoses are genetically determined cornification disorders of the epidermis. They are characterized by the presence of different degrees of scaling, hyperkeratosis, and erythroderma, often associated with palmoplantar keratoderma (PPK) [1]. These classes of diseases are caused by a variety of mutations in genes involved in epidermal development and skin barrier maintenance [2]. In recent decades, several of these inherited skin diseases have been molecularly characterized [3,4,5,6,7]. Different classifications of these diseases have been proposed, often based upon the involved genes and/or clinical presentation. The current classification [8] divides these clinical conditions into syndromic and non-syndromic disorders. The syndromic ichthyoses refer to conditions in which the disorder is part of a more complex syndrome, and the genetic defect affects other organs as well. In non-syndromic ichthyosis, the manifestation of the underlying genetic abnormality is confined to the skin district. The group of non-syndromic ichthyoses includes autosomal recessive congenital ichthyosis (ARCI), which is clinically represented as lamellar ichthyosis (LI), congenital ichthyosiform erythroderma (CIE), or harlequin ichthyosis (HI). The latter is a rare and severe form of ichthyosis, which may be fatal. Newborns can be enclosed in a shell of thick scale plates with deep fissures [9]. In this disease, the skin barrier is severely compromised, leading more susceptible newborns to sepsis and dehydration [10,11]. Somatic mutations generating a blaskoid mosaic of the disease have also been described [12]. According to this classification, epidermis diseases with an ichthyosis phenotype caused by keratin mutations are now included in the keratinopathic ichthyosis (KPI) group, while disorders with epidermolysis are included in the epidermolytic ichthyosis (EI) group. Eritrokeratodermia variabilis (EKV, OMIM 133200) is another peculiar disease that is caused by defects in the process of keratinization. EKV is a rare, congenital, epithelial disease with early onset, characterized by remitting–relapsing erythematous patches associated with plaques of hyperkeratosis, sometimes massive (hystrix-like), stationary, or migratory [13]. The EKV disease has been associated with mutations in connexin genes *GJB3* (*Cx31*) [14] and *GJB4* (*CX30.3*) [15,16]. Recently, a variant of this disease has been identified, caused by mutations in connexin gene GJA1 (*Cx43*) [17]. Since it has not been recognized as a distinct entity, the new definition of erythrokeratodermia variabilis et progressiva (EKVP) has now been used to include the *GJA1* variant. The accurate prevalence of EKVP is unknown, but it is estimated to be less than 1:200.000, being primarily inherited as an autosomal dominant connexin-dependent disease. Despite its dominant transmission, few cases of recessive transmission have been reported [18,19,20,21].

The clinical features of EKVP present some overlap with other forms of ichthyoses such as lamellar ichthyosis or epidermolytic hyperkeratosis. Indeed, EKVP can be included within the ichthyosis group of mendelian disorders of cornification (MeDOC) [8], although its clinical phenotype is not characterized by generalized scaling of the epidermis and/or erythema. In addition, EKVP presents histologically with a thinner stratum corneum in respect of the other types of ichthyoses.

Although some of the genetic bases of EKVP have been discovered, the mechanism by which the reported mutations in connexin genes give rise to the general phenotype of the disease is still unclear.

To date, the connexin genes identified as causative of the disease have been found to be mutated in about 60–65% of cases (http://www.ichthyosis.org.uk; accessed on 2 February 2021) [8,16], and other genes responsible for the EKVP phenotype in these families have not been characterized yet. Although the first genetic cause was identified several years ago, many patients do not show connexin mutations, indicating further unknown genetic heterogeneity. In this study, we analyzed a family composed of five individuals with two of the three siblings affected by a dermatological disease in which the clinical phenotype suggests a form of EKVP.

## 2. Results

### 2.1. Patient Presentation

**Figure 1:**
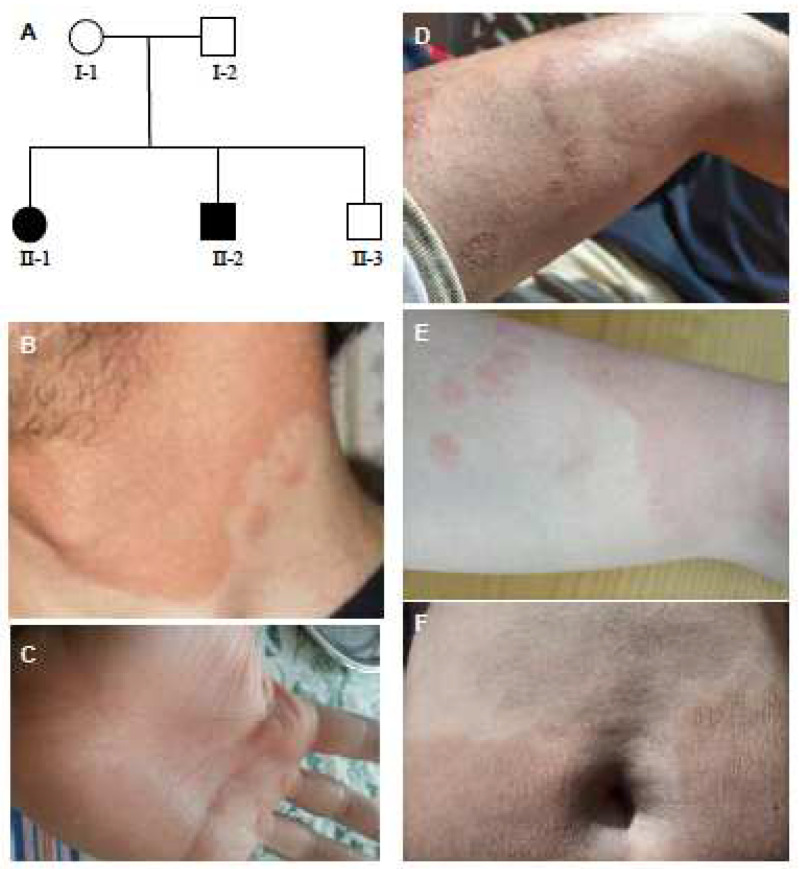
Family pedigree and clinical presentation. (**A**) Family pedigree; (**B**–**D**) Skin ichthyosis, erythroderma and palmar keratoderma of the male patient. (**E**,**F**) Clinical presentation of the female patient.

Five individuals comprised the family studied in this work: two parents and three siblings. Two siblings, a 27-year-old male and a 32-year-old female, were affected by dermatological lesions (Figure 1A), whereas the 41-year-old male did not present with any skin lesion.

The male patient presented with erythematous patches on body skin, which changed in size and shape over the weeks (Figure 1B). His lesions were predominantly in the neck region and over both superior and inferior limbs. Hyperkeratosis of palms and soles (Figure 1C) was also detected. Light white scales also characterize the ichthyotic hyperkeratosis. The clinical presentation of the female patient was similar to her male sibling, although the ventral region, the torso, and the back, in addition to the neck region and limbs, were the most affected areas (Figure 1E,F). Various small areas of skin were unaffected in both patients. It is interesting that some lesions appeared with erythema and scaling, while other lesions presented only as eczematous patches (Figure 1D,E). In both cases, lesions always tended to originate in a symmetrical fashion following a point-to-patch evolution starting from extensor surfaces and the inside of cavities such as axillae and elbows. The patients’ parents recalled the presence, at birth, of erythema localized only on the limbs, which then disappeared after around one week in both cases. The erythematous lesions then reappeared at six months of age in both patients, spreading throughout the body for days and migrating for months, creating geographical patterns. During the first decade of the female patient’s life, the erythema component decreased in intensity while the diffusion of hyperkeratotic areas increased. The opposite happened in the male patient, whose patches of thickened skin tended to remain smaller but more erythematous. No other family members, including grandparents and a father-side aunt, were affected by the disease, nor by any other relevant dermatological pathology.

### 2.2. Histological Examination

**Figure 2:**
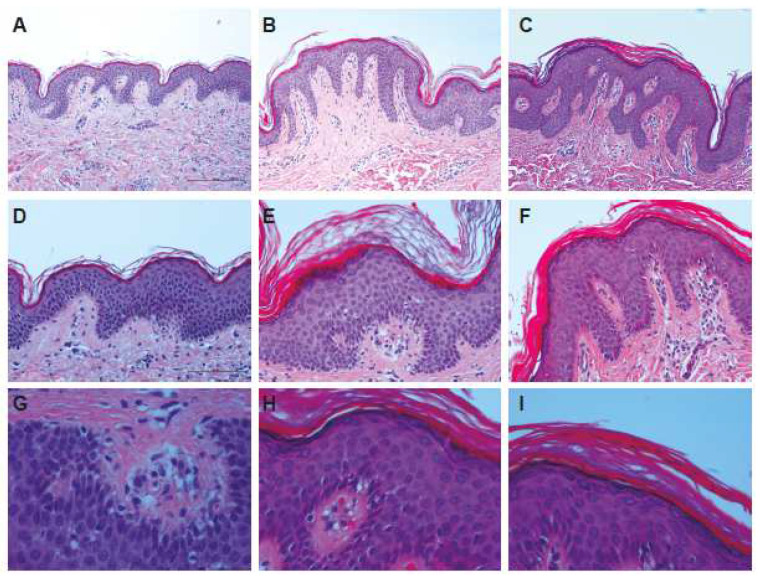
Histological analysis. (**A**) Normal skin; (**B**) skin biopsy from II-1 patient; and (**C**) skin biopsy from II-2 patient (10× magnification); (**D**) normal skin; (**E**) skin biopsy from II-1 patient; and (**F**) skin biopsy from II-2 patient (20× magnification). (**G**) Details of II-1 biopsy; (**H**,**I**) details of II-2 biopsy (40× magnification).

The histological examination of patients’ sections clearly shows the presence of epidermal hyperplasia without signs of epidermolysis (Figure 2B,C) with respect to the normal control (Figure 2A). Higher magnifications show ortho-hyperkeratosis and evident papillomatosis that in some cases includes portions of derma (Figure 2C). Light acantholysis and superficial lymphocyte infiltration above the granular layer (Figure 2E,F) are additionally visible. Furthermore, detailed histological examination shows patches of vacuolization in the basal layer (Figure 2G), and hyperkeratosis with a characteristic basket conformation typical of EKVP in the stratum corneum is clearly visible (Figure 2H,I).

To further characterize the epidermal modifications present in our patients, we performed an immunofluorescence analysis using specific fluorescent antibodies and confocal microscopy to highlight the distribution of specific epidermal proteins fundamental in skin physiology and differentiation.

**Figure 3:**
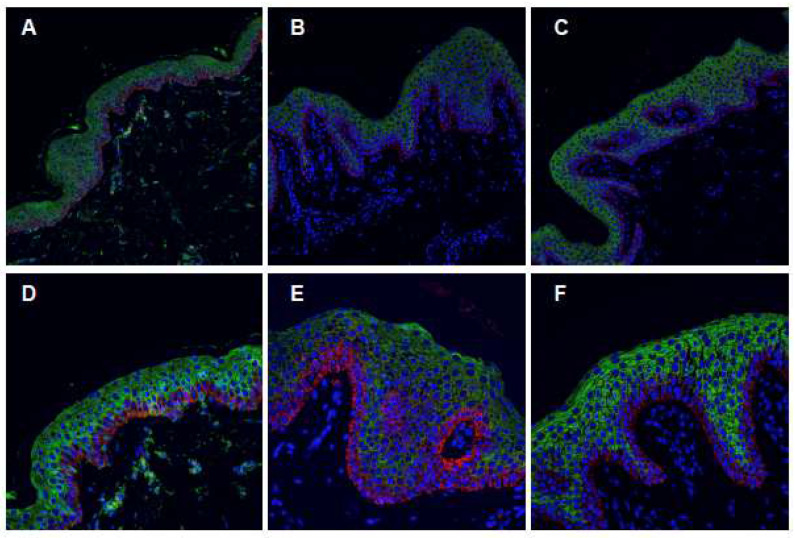
Immunofluorescence confocal analysis. (**A**) Normal skin; (**B**) skin biopsy from II-1 patient; and (**C**) skin biopsy from II-2 patient (20× magnification). (**D**) Normal skin; (**E**) skin biopsy from II-1 patient; and (**F**) skin biopsy from II-2 patient (40× magnification). Green, KRT10; Red KRT14; and Blue, nuclei (DAPI).

The skin sections were stained using antibodies directed against KRT14 and KRT10. The results show a normal basal compartment, composed of a single-cell layer (Figure 3B,C, red stain) similar to the layer observable in the control normal skin (Figure 3A). The distribution of KRT10, however, showed the thickening of the suprabasal layer (Figure 3A–C, green staining), especially in areas of hypertrophic papillae (Figure 3B,C, stars). This feature is particularly evident in higher magnification pictures (Figure 3E,F). It is important to note that the keratin cytoskeleton appears normal in both basal and suprabasal layers, since no clumping of filaments is visible. The distribution of the two keratins is regular within the keratinocyte cytoplasm.

**Figure 4:**
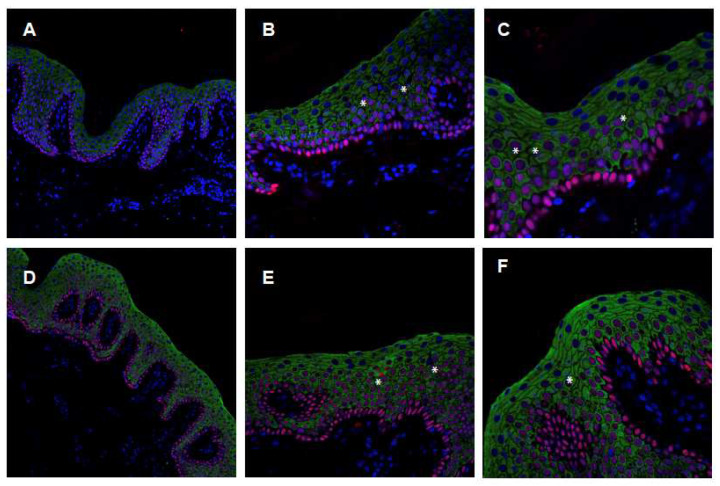
Immunofluorescence confocal analysis, p63 KRT1 staining. (**A**–**C**) Skin biopsy from II-1 patient (20×, 40×, 60× magnification); (**D**–**F**) skin biopsy from II-2 patient (20×, 40×, 60× magnification). Asterisks (*) highlight positive p63 nuclei in the suprabasal layer. Green, KRT1; Red, p63; and Blue, nuclei (DAPI).

An important marker of keratinocyte proliferation is represented by the transcription factor p63, belonging to the p53 family [22]. Cells actively expressing p63 retain a proliferative potential and are considered like stem cells. They lead to the constant renewal of the epidermis and mutations in the p63 gene are the cause of specific genodermatoses [23,24]. In skin diseases with a high rate of cell proliferation, positive p63 cells are identified outside the basal layer. The staining of patient skin sections with anti-p63 antibodies (Figure 4) shows (Figure 4B,C,E,F) the presence of positive nuclei (red) in the upper epidermal layers in respect of the basal layer.

A few p63-positive nuclei (red) in association with positive KRT1 staining (green) are visible as well. Keratinocytes within the suprabasal layer are visible (Figure 4B,C,E,F) that are positive for both KRT1 and p63 (stars in Figure 4), thus indicating an increased proliferation index.

### 2.3. Genetic Analysis

**Figure 5:**
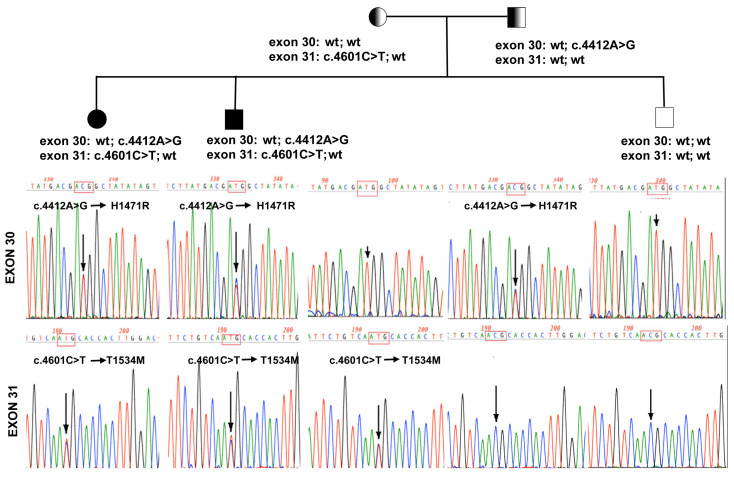
Genetic analysis. This figure reports the family pedigree (upper part) and the sequence electropherogram of part of *ABCA12* exon 30 (Upper row of boxes) and *ABCA12* exon 31 (lower boxes). Each box is related to a family member, and in columns, the sequence of exon 30 and 31 for each patient is shown.

A genetic analysis was performed to identify the molecular defects that cause the epidermal hyperproliferation, ichthyosis, and erythema characterizing the patients’ phenotype. Patients’ pedigree shows two of the three siblings affected by the described genodermatosis, while the parents were unaffected. The clinical findings were initially consistent with the diagnosis of EKVP. Since our group and other investigators described homozygous mutations in connexin genes [18,20,21], we firstly sequenced and analyzed genes for *GJB3*, *GJB4*, *GJB5*, and *GJA1*, without detecting any mutation. We performed audiometric analysis on the probands, who did not reveal any degree of hearing impairment. The patients were re-evaluated to assess the diagnosis both clinically and histologically. Since the phenotype of patchy erythema and ichthyosis was already described in the literature [25,26], as correlated to mutations in *KRT1* and *KRT10* genes, we sequenced these genes as well, but no mutations were found. Then, we performed exome next-generation sequencing (NGS) analysis of the whole family revealing several genomic variations, but no new common gene causative for EKVP was found. However, filtering the variations of genes responsible for epidermal phenotypes, we found two different heterozygous mutations segregating with the family disease (Figure 5). The mutations are located in exon 30 and exon 31 of the *ABCA12* gene, the major gene associated with harlequin ichthyosis [27].

The first variation was identified by position NM_173076.3:c.4412A>G, leading to the aminoacidic substitution p.H1471R, and to the codon change from H [CAT] to R [CGT] in exon 30. This variation is identified by the Reference SNP (rs) rs144220620. The substitution is predicted to be deleterious by SIFT, REVEL, and MetaLR. Furthermore, the analysis of allele frequencies reveals a rare allele (TOPMed reports an allele frequency of T: 0.999984072 and C: 1.59280 × 10^−5^, and currently there are not citing references or clinical variants).

The second variation was the missense NM_173076.3:c.4601C>T, leading to the substitution p.T1534M, altering the original codon from T [ACG] to M [ATG] in exon 31. This variation is identified as rs200407397. The analysis of allele frequencies showed the mutated allele C = 0.000023 (6/264690, TOPMED) and C = 0.000004 (1/251170, GnomAD_exome). The prediction of this mutation is deleterious according to SIFT. No citing references are available for this variation.

The distribution of the variation in the family showed that the affected siblings are double heterozygous for the described mutations, while the unaffected sibling shows a wild-type genotype. The parents are each heterozygous for one of the mutations identified. The segregation of the two variations demonstrates that they are present in two distinct alleles.

In conclusion, the disease that affects the two patients could be caused by the simultaneous presence of the two mutations in a double heterozygous fashion.

### 2.4. Characterization of the ABCA12 Physiological Modifications

Since specific literature regarding the two mutations identified was not available, we investigated the possible mechanism by which these mutations can contribute to the pathogenesis of the disease in the patients studied. Both mutations p.H1471R and p.T1534M are located within the 1350–1565 cytoplasmic domain that contains important residues for ATP binding and hydrolysis, a domain that tends to be conserved in this class of transporters’ lipid family.

**Figure 6:**
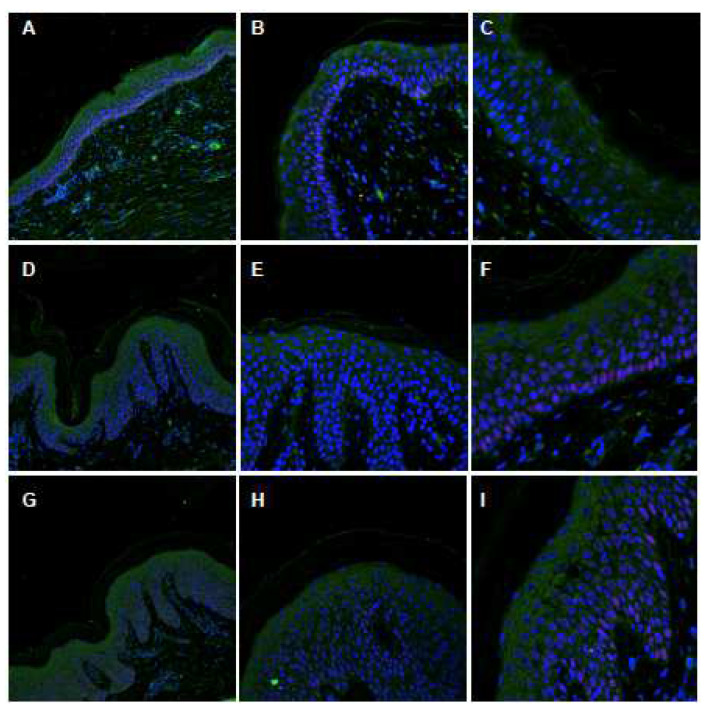
Immunofluorescence confocal analysis, ABCA12 staining. (**A**–**C**) Normal skin biopsy (20×, 40×, 60× magnification). (**D**–**F**) Biopsy from II-1 patient (20×, 40×, 60× magnification). (**G**–**I**) Skin biopsy from II-2 patient (20×, 40×, 60× magnification). Green, KRT1; Red, p63; and Blue, nuclei (DAPI).

The presence of mutations can affect RNA synthesis as well as protein translation, even if they are not a stop codon or large deletion [10], leading in some cases to a depletion of the protein in the epidermis of affected patients. To investigate whether the gene expression of *ABCA12* is affected by the presence of mutations, we firstly performed an immunofluorescence staining experiment using skin sections from patients’ affected areas, and from a healthy control. The presence of ABCA12 in the normal control appears cytoplasmic, with low expression in the basal layer and then increasing in the spinous and granular layers (Figure 6A), in accordance with other published data [28]. High magnification images confirmed this distribution and localization in the control skin (Figure 6B,C). The staining with an ABCA12 antibody demonstrates a normal ABCA12 protein distribution in both patients (Figure 6D–I), with the maximum intensity at the level of the spinous and granular layer. Also in this case, high magnification confirmed cytoplasmic expression (Figure 6F,I).

Since the biochemical activity of ABCA12 consists of the transfer of glucosylceramides (GlcCer) to the extracellular environment, where specific enzymes located in the intercellular spaces hydrolyze these molecules to ceramides, we tried to verify the effects of mutations on ABCA12 activity in the patients under examination through the analysis of glucosylceramide distribution.

To achieve this objective, we performed further immunofluorescence experiments and confocal analysis on patients’ skin sections using a specific anti-GlcCer antibody. This analysis allowed us to obtain important data on glucosylceramide secretion in the upper part of the granular layer of the two patients compared to the healthy control.

**Figure 7:**
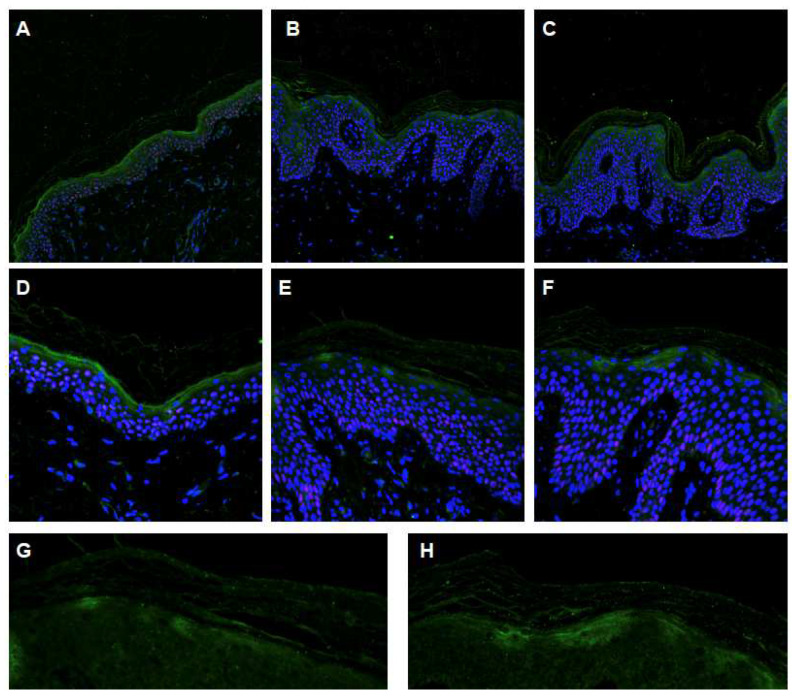
Immunofluorescence confocal analysis, GlcCer staining. (**A**,**D**) Normal skin biopsy (20×, 40× magnification). (**B**,**C**) Biopsy from II-1 and II-2 patients (20× magnification). (**E**,**F**) Skin biopsy from II-1 and II-2 patients (40× magnification); (**G**,**H**) details at 60× magnification of GlcCer patchy distribution in both patients. Green, GlcCer; Blue, nuclei (DAPI).

GlcCer in the normal control (Figure 7A) is mainly located in upper skin layers. At high magnification, it was possible to show its presence mainly in the granular layer (Figure 7D), where it forms a continuous band under the stratum corneum. We also identified a signal within the stratum corneum but limited to the contact between corneocytes (Figure 7C). Patient sections showed a clear lower intensity of the green signal, identifying the presence of GlcCer (Figure 7B,C). Furthermore, at high magnification (Figure 7E,F) it is evident that GlcCer is not only under-represented with respect to the control, but it does not form a continuous band as in the control sections either. Further enlargement and analysis of the GlcCer staining confirmed the patchy presence of ceramide in the stratum granulosum of patients’ skin (Figure 7G,H).

The last experiment demonstrated a relationship between the presence of nucleotide variations and the impairment of ABCA12 physiological function. From the data we gathered, it is evident that the activity of the enzyme is impaired, but not totally abolished in these two patients. Indeed, it is still possible to retrieve the GlcCer even in a lower concentration and in a patchy pattern. At this point, we can speculate that the substitutions H1471R and T1534M could be considered the causative mutations of the patients’ phenotype.

## 3. Discussion

The main role of the ABCA12 enzyme within the epidermis is to transports lipids such as glucosylceramides from the outer to the inner leaflet of lamellar granule (LG) membranes. The lipids are transported to the keratinocyte periphery via the trans-Golgi network and released to the apical surface of the granular keratinocytes where they contribute to the final impermeabilization of the stratum corneum, which is essential for skin barrier function [10,29]. The loss of this chemical/physical barrier leads to an impaired barrier function, indicated by an increased trans-epidermal water loss (TEWL) and decreased water-binding capacity. In addition, this impaired barrier leads to an increased access of contaminants such as small molecules, viruses, and bacteria [30]. During this pathological process, keratinocytes synthesize and secrete inflammatory mediators such as prostaglandins, eicosanoids, leukotrienes, histamines, and cytokines, which in turn lead to inflammatory responses within the skin [31]. The presence of hyperkeratosis should be ascribed to the impairment of the barrier function, as the skin reacts with a compensatory hyperproliferation of keratinocytes, hence leading to hyperkeratosis [32]. The molecular data presented in this work show a partial depletion of the ABCA12 function, with a reduced and patchy deposition of the hydroxyceramides. The reported patients’ phenotype with erythema and hyperkeratosis can be associated with the inflammation and hyper-keratinization of the skin due to an impaired epidermal barrier associated with the ABCA12 activity reduction.

This case report confirms that mutations in the *ABCA12* gene could not only cause the rare skin condition called harlequin ichthyosis, but they might also lead to a phenotype that is attributable clinically to the much less severe EKVP disease. Importantly, in this study, we clarify that the severity of the disease is related to mutations that impair the function of ABCA12. The two specific mutations identified do not completely abolish ABCA12 activity as evidenced by the patchy presence of glucosyl-ceramide in the epidermis, thus giving rise to an intermediate phenotype resembling EKVP. In addition to genetic and histological characterizations, it is relevant to report that, when the clinical diagnosis of EKVP was carried out (not long before our investigation), the two affected patients were started on a topical treatment that included isotretinoin 0.05%, a well-known teratogenic compound. This topical treatment led to the desquamation and to the almost complete resolution of the skin lesions in the male subject, whereas it only improved the clinical appearance of the lesions in the female subject. The different responses to the treatment could be attributable to the conservative approach in the dosage and the use of isotretinoin in the female patient, who was still at an age of child-bearing potential. This information about this genetic condition could foster the interest in the field of research on the use of isotretinoin for the treatment of rare and sometimes life-threatening skin conditions.

## 4. Materials and Methods

### 4.1. Genetic Analysis

Total RNA extraction from biopsy of the left dorsal forearm was performed using RNeasy minikit (Qiagen, Crawley, UK). Reverse transcription was performed through Superscript II Reverse Transcriptase (Invitrogen, Carlsbad, CA, USA). Genomic DNA of patients was extracted from peripheral blood using Wizard^®^ Genomic DNA Purification kit (Promega, Madison, WI, USA), according to manufacturer’s instructions.

The following primers were used for keratin amplification and sequence of *ABCA12* exon 30 and 31: ABCA12 Exon 30FW 5′-GCACAAAATAGGTAGGAACTC-3′, Exon 30REV 5′-ATTATTGACAGCGTCTCAC-3′. ABCA12 Exon31FW 5′-TACAGGCGTGAACCAGTGAG-3′, and Exon31REV 5′-CAGTCCTCAGACCAGCAACA-3′.

For *KRT9* amplification and sequence, we used primers as in [18,33], for *KRT16* and *KRT6a* refer to references [34,35,36], and for *KRT10* and *KRT10* refer to [37]. For the reverse transcription and PCR amplification, total RNA (1 μg) was used for reverse transcription reaction with SensiFAST^TM^ cDNA Synthesis kit (ThermoFisher Scientific, Waltham, MA, USA). Amplification was performed using fusion high-fidelity Taqman (ThermoFisher Scientific, USA).

PCR products were purified with QIAquick^®^ Gel Extraction kit (Qiagen, UK) and directly sequenced. Cloning was performed using pcr2.1 vector (TA Cloning ^TM^ kit, ThermoFisher Scientific, USA) and DH5**α** competent bacteria according to standard protocols. Digestion Analysis BstXI (New England Biolabs, Hitchin, UK, #R0113S) has been performed using 1 mg of vector DNA, in a total volume of 50 mL using 0.5 mL of enzyme in 1× NEBuffer™ r3.1, incubated at 37 °C for 1 h.

#### Exome Sequencing

Libraries for whole exome sequencing have been prepared using the Illumina Nextera Expanded Exome Enrichment Kit, containing more than 62 Mb of genomic content, including exons, UTRs, and miRNA. Libraries have been sequenced on the Illumina Hiseq2000 sequencer and analyzed with the Illumina extraction pipeline. The target average coverage was greater than 70×. The activity has been performed as a service at CRS4, Pula (CA), Italy.

### 4.2. Light Microscopy

Patient and control skin biopsies were analyzed with light microscopy, through hematoxylin-eosin staining according to standard methods.

### 4.3. Confocal Immunofluorescence Analysis

Skin sections were fixed in formalin 4%, permeabilized with Triton X-100 0.1% in PBS 1× and embedded in paraffin. Sections were incubated in heater for 1 h, washed in Bio-Clear (Bio-Optica, Milano, Italy) to remove paraffin, and rehydrated with decreasing alcohol concentration washes (100, 95, 80, 70, 50% H_2_O, Sigma Aldrich, Gillingham, UK). After boiling in sodium citrate (0.01 M, pH 6.0) for antigen unmasking, sections were stored overnight in sodium tetrahydroborate (NaBH4, Sigma Aldrich, Burlington, MA, USA) at 4 °C. After incubation in blocking buffer (PBS1X + 5% goat serum) for 2 h at room temperature, the following primary antibodies were used: mouse polyclonal anti-K14 (LL02, Abcam, dilution 1:1000), rabbit polyclonal anti-K1 (Covance, dilution 1:1000), mouse polyclonal anti-p63 (Abcam Ab735, dilution 1:500), rabbit anti-ABCA12 (H-300) (Santa Cruz Biotechnology Dilution 1:100), and rabbit anti-GlcCer (Glycobiotech dilution 1:100). The secondary antibodies were Alexa fluor^®^488 goat anti-rabbit IgG (H + L) (Invitrogen, Carlsbad, CA, USA, dilution 1:1000) and Alexa fluor^®^568 goat anti-mouse IgG (H + L) (Invitrogen, Carlsbad, CA, USA, dilution 1:1000). Nuclei staining was performed by DAPI (ThermoFisher Scientific, Waltham, MA, USA, 5 mg/mL stock solution, used at dilution 1:1000). All antibodies were prepared in blocking buffer. Sections were covered by Prolong Antifade reagent (Invitrogen, USA). Images of section were obtained using a confocal laser microscope Nikon Eclipse Ti. Laser at 405 nm was used for DAPI detection, a laser of 561 nm for the detection of Alexa fluor^®^568 and 488 nm for Alexa fluor^®^488. Signal analysis was performed using NIS Element AR (Nikon) software, version 4.00.04.

## Data Availability

The data supporting this study’s findings are not publicly available due to the need to protect patient privacy, but are available on reasonable request from the corresponding author, A.T.
